# Sociodemographic aspects, time series and high-risk clusters of malaria in the extra-Amazon region of Brazil: a 22-year study

**DOI:** 10.1590/0037-8682-0564-2023

**Published:** 2024-11-08

**Authors:** Rosália Elen Santos Ramos, Erica Santos dos Reis, Leticia Pereira Bezerra, Maria Wilma da Silva Lima, Ana Paula Sampaio Feitosa, Luiz Carlos Alves, Israel Gomes de Amorim Santos, Fábio André Brayner

**Affiliations:** 1 Universidade Federal de Pernambuco, Centro de Ciências Médicas, Recife, PE, Brasil.; 2 Universidade Federal de Sergipe, Departamento de Medicina, Aracajú, SE, Brasil.; 3 Instituto Aggeu Magalhães - FIOCRUZ, Departamento de Parasitologia, Recife, PE, Brasil.; 4 Universidade Estadual de Alagoas, Departamento de Biologia, Santana do Ipanema, AL, Brasil.

**Keywords:** *Plasmodium* spp, Neglected Disease, Spatial analysis, Epidemiology, Public Health

## Abstract

**Background:**

Malaria is an acute febrile parasitic disease that significantly impacts global public health. In Brazil, the most studied endemic area for the disease is the Amazon region. This study aims to analyze temporal, spatial, and spatiotemporal patterns of malaria in the extra-Amazon region of Brazil over a 22-year period.

**Methods:**

We conducted a time-series study from 2001 to 2022, encompassing both autochthonous and imported cases. Time trend analysis was employed to assess fluctuations in incidence rates over the years. Spatial clusters of infection risk were identified using the Local Moran Index and Kulldorff's scan.

**Results:**

A total of 18,633 malaria cases were identified in the extra-Amazon region, including 1,980 autochthonous, 13,836 imported, and 2,817 of unknown origin. During the first period (2001-2011), 1,348 autochthonous and 9,124 imported cases were reported. In the second period (2012-2022), there were 632 autochthonous and 4,712 imported cases. The state of Espírito Santo exhibited a decreasing trend but maintained the highest incidence rates throughout the study. The number of municipalities at high risk for autochthonous cases declined, with Espírito Santo, Minas Gerais, and Piauí having the most municipalities with high rates. For imported cases, the federative units with the highest numbers in both periods were Ceará, Distrito Federal, Goiás, Minas Gerais, Piauí, and Paraná.

**Conclusions:**

The data reveal the areas most affected by malaria and thus of highest priority for implementing control strategies.

## INTRODUCTION

Malaria is an acute febrile disease caused by protozoa of the genus*Plasmodium*, transmitted to humans through the bite of female*Anopheles*mosquitoes. Recognized by the World Health Organization as a life-threatening condition, malaria remains a major public health challenge. In 2021, there were 247 million cases and 627,000 deaths globally, with half of the world's population at risk of infection^1^ ^,^ ^2^.

In Brazil, the predominant malaria species are*Plasmodium vivax*and*Plasmodium falciparum*.*P. vivax*accounts for 83.7% of the cases reported; despite its lower lethality, its high incidence results in mortality rates comparable to those of*P. falciparum*. In 2022, Brazil reported 131,224 malaria cases. In response, the Ministry of Health launched the National Malaria Elimination Plan, targeting the eradication of the disease by 2035^3^ ^,^ ^4^.

More than 99% of malaria cases in Brazil are autochthonous and occur in the Amazon region^5^. In the extra-Amazon region, despite low incidence, all states reported at least one autochthonous case between 2010 and 2021, except Sergipe. Espírito Santo reported a higher frequency of autochthonous than imported cases, with significant case percentages also in Bahia and São Paulo, and the highest incidence rates in Piauí and Paraná^6^ ^,^ ^7^. Despite the low number of cases, the mortality rate in this region exceeds that of the Amazon, largely due to delayed diagnosis and treatment stemming from a lack of awareness among healthcare professionals^6^ ^,^ ^8^. 

Spatial analysis tools are pivotal in understanding the variation and spread of malaria^9^ and assessing the effectiveness of control strategies implemented by health services^10^. The identification of new cases and clusters using spatial statistics is critical in prioritizing transmission control measures, which in turn helps to improve population health and reduce the risk of malaria^11^ ^-^ ^13^. In Brazil, these tools are essential for effective surveillance, diagnosis, and antimalarial treatment policies, all of which are crucial for achieving the goals of the National Malaria Elimination Plan. 

Therefore, this study aims to analyze the behavior of malaria cases in the extra-Amazon region of Brazil over 22 years, both prior to and during the implementation of the National Malaria Elimination Plan. It also seeks to describe the epidemiological scenario and examine the temporal, spatial, and spatiotemporal patterns of malaria during this period. 

## METHODS

### ● Study design

An ecological time-series study utilizing spatial analysis tools was conducted from 2001 to 2022. The study included both autochthonous and imported malaria cases identified in the federation units of the extra-Amazon region of Brazil. The units of analysis encompassed all municipalities within this region. Autochthonous cases are those where an individual becomes infected in their own area of residence where malaria transmission is ongoing (local transmission). Imported cases refer to individuals who acquire the disease in endemic areas but are diagnosed in regions where there is no continuous local transmission. Cases identified outside the Legal Amazon are categorized as extra-Amazonian malaria cases^3^.

### ● Study area

The federation units comprising the extra-Amazon region of Brazil, which are outside the boundaries of the Legal Amazon, include: Alagoas (AL), Bahia (BA), Ceará (CE), Federal District (DF), Espírito Santo (ES), Goiás (GO), Mato Grosso do Sul (MS), Minas Gerais (MG), Paraíba (PB), Paraná (PR), Pernambuco (PE), Piauí (PI), Rio de Janeiro (RJ), Rio Grande do Norte (RN), Rio Grande do Sul (RS), Santa Catarina (SC), São Paulo (SP), and Sergipe (SE)^9^. In 2022, these states had an estimated population of 175,290,524, spread over a total area of 3,426,970.715 km², representing 40% of Brazilian territory. Together, these states account for 91.6% of the country’s Gross Domestic Product^14^. Recommended diagnostic practices in these states include the use of rapid tests, which have low sensitivity. Notifications of suspected cases must be reported within 24 hours^15^.

### ● Data source

The data on autochthonous and imported cases per year, as well as sociodemographic characteristics, were sourced exclusively from the Notifiable Diseases Information System (SINAN) database maintained by the Department of Informatics of the Unified Health System (DATASUS), which is provided by the Brazilian Ministry of Health. Cure verification slides (CVSs) were excluded from this analysis. Additionally, population data and the digital cartographic grid (in shapefile format), segmented by municipalities and states according to the Universal Transversal Mercator (UTM) system and the horizontal Terra Datum model (SIRGAS 2000), were obtained from the Brazilian Institute of Geography and Statistics (IBGE). This study utilized population data from the 2010 demographic census and intercensal estimates. 

### ● Variables and measures

The primary variable of this study was the malaria incidence rate (per 100,000 inhabitants), calculated at the municipal level. Crude rates were calculated for segmented periods within the analysis timeframe to better understand temporal variations in incidence rates, from 2001 to 2011 (Period 1, P1) and 2012 to 2022 (Period 2, P2). The division of the study years into two periods facilitated a more nuanced understanding of the dynamics of the disease over time and within the context of Brazil, before and after the implementation of the National Malaria Elimination Plan. To enhance standardization, each period covered eleven years. The incidence rate was calculated using the formula: (number of cases divided by the mean population of the period) multiplied by 100,000. Additionally, we calculated both the absolute and relative frequencies of variables such as sex, age group, race/color, education level, and results of parasitological examinations. 

### ● Temporal trend analysis

Temporal trend analysis was conducted for the autochthonous cases using segmented linear regression with the Joinpoint Regression Program, version 4.9.0.0. We calculated crude incidence rates for the study area population and by federation units to assess temporal trends. This method allowed us to detect changes in the trend of the variables over time, fitting the data into a time series with the minimal number of junction points. Accordingly, the time series could exhibit an increasing, decreasing, or stable trend^16^.

We utilized the Monte Carlo permutation test to select the optimal segment for each model, employing 9,999 permutations. Additionally, we calculated the annual percentage change (APC) for each period and the average annual percentage change (AAPC) across the entire period when more than one significant inflection point was identified^17^. Temporal trends were considered statistically significant if the APC and AAPC showed a p-value <0.05 with a 95% confidence interval (CI).

### ● Spatial and spatiotemporal analysis

Maps illustrating the crude incidence rates of autochthonous malaria cases for P1 and P2 in the extra-Amazon region were created. We then applied the Local Empirical Bayesian method to smooth the rates, correcting for random fluctuations and enhancing the stability of the values obtained. Both crude and smoothed incidence rates were categorized according to guidelines from the Brazilian Ministry of Health^3^ as follows: very low (<1.0/100,000 inhabitants), low (1.0 to 9.0/100,000 inhabitants), moderate (10.0 to 49.9/100,000 inhabitants), and high (≥50.0/100,000 inhabitants).

Following da Paz et al.^17^, we assessed spatial autocorrelation using the Global Moran Index (GMI), which ranges from -1 to +1, indicating the correlation of a variable with itself. Using the Local Moran Index (Local Indicators of Spatial Association - LISA), we examined local spatial autocorrelation to identify municipalities with similar patterns through clusters of high and low risk and transition, resulting in four quadrants: Q1 (high/high), Q2 (low/low), Q3 (high/low), and Q4 (low/high). The first two quadrants represent municipalities with similar values to their neighbors, while the last two represent municipalities with dissimilar values from their neighbors and no spatial association^18^. A significance level of 0.05 was used. These analyses were performed using TerraView software, version 4.2.2, and maps were constructed with QGIS software, version 3.18. 

Continuing with methods from da Paz et al.^17^, we employed spatiotemporal scan statistics following Kulldorff’s retrospective analysis method to detect high-risk clusters using a Poisson probability model. The conditions for the cluster analysis included an aggregation time of 1 year, no overlapping clusters, circular clusters, a maximum spatial cluster size of 10% of the population at risk, and a maximum temporal cluster size of 50% of the study period^19^. 

Primary and secondary clusters were identified using the log-likelihood ratio (LLR) test, with the highest LLR indicating the most likely cluster. These are represented in maps and tables. Relative risks (RR) were calculated for each cluster compared to its neighbors, with results deemed significant at a p-value <0.05 based on 999 Monte Carlo simulations^19^. Analyses were conducted using SatScan software, version 10.0.2, and maps were produced using QGIS software, version 3.18. 

Finally, maps depicting the absolute number of imported cases were created, categorized according to the Brazilian Ministry of Health standards with adaptations for different thresholds: >5 cases, 5 to 24 cases, 25 to 49 cases, and ≥ 50 cases.

## RESULTS

From 2001 to 2022, a total of 18,633 malaria cases were reported in the extra-Amazon region of Brazil, comprising 1,980 autochthonous (Supplementary Material 1), 13,836 imported (Supplementary Material 2 **),** and 2,817 uncategorized cases. During P1 (2001-2011), there were 1,348 autochthonous and 9,124 imported cases notified, whereas P2 (2012-2022) saw 632 autochthonous and 4,712 imported cases. Table 1 shows that the predominant sociodemographic characteristics among malaria cases in both periods were male, aged between 20 and 39 years, of white race/color, individuals with primary education, and those diagnosed with*P. vivax*.

**TABLE 1:** Sociodemographic characteristics of malaria cases in the extra-Amazon region, Brazil, 2001-2011 (P1), 2012-2022 (P2) and 2001 to 2022.

Variables	P1						P2						2001 to 2022					
	N	Population	%	Incidence*	Imported	%	N	Population	%	Incidence*	Imported	%	N	Population	%	Incidence*	Imported	%
**Sex**																		
Female	557	83,140,024	29.4	0.7	1,744	19.0	318	91,509,909	31.1	0.3	1,100	557	875	87,324,967	30.0	1.0	2,844	20.0
Male	1,335	79,552,905	70.6	1.7	7,461	81.0	703	86,972,093	68.9	0.8	3,911	1,335	2,038	83,262,499	70.0	2.4	11,372	80.0
**Age group (years)**																		
< 1	13	2,544,730	0.7	0.5	36	0.4	11	2,434,780	1.1	0.5	31	0.62	24	2,489,755	0.8	1.0	67	0.5
1 - 19	464	54,418,384	24.5	0.9	324	3.7	151	48,663,947	14.8	0.3	341	6.81	615	51,541,166	21.1	1.2	665	4.8
20 - 39	739	53,986,472	39.0	1.4	2,540	28.9	407	58,359,328	39.9	0.7	2,431	48.51	1,146	56,172,900	39.3	2.0	4,971	36.0
40 - 59	527	35,480,956	27.8	1.5	3,792	43.1	343	44506980	33.6	0.8	1,788	35.68	870	39,993,968	29.9	2.2	5,580	40.4
≥ 60	150	16,262,387	7.9	0.9	2,095	23.8	109	24,516,967	10.7	0.4	420	8.38	259	20,389,677	8.9	1.3	2,515	18.2
Missing data	0	-	0.0	0	2	0.0	0	-	0.0	0	0	0.0	0	-	0.0	-	2	0.0
**Race/Color**																		
White	805	868,79899	42.5	0.9	4,352	47.0	354	86,478,562	34.7	0.4	2,035	40.6	1,159	86,679,231	39.8	1.3	6,387	44.9
Black	82	11,322,553	4.3	0.7	550	6.0	111	15,846,351	10.9	0.7	516	10.3	193	13,584,452	6.6	1.4	1,066	7.5
Yellow	15	837,781	0.8	1.8	109	1.0	5	919,296	0.5	0.5	54	1.1	20	878,539	0.7	2.3	163	1.1
Brown	622	64,037,462	32.8	1.0	2,771	30.0	473	73,400,293	46.3	0.6	1,924	38.4	1,095	68,718,878	37.6	1.6	4,695	33.0
Indigenous	108	406,075	5.7	26.6	64	1.0	12	502,920	1.2	2.4	19	0.4	120	454,498	4.1	26.4	83	0.6
Missing data	262	-	13.8	-	1,362	15.0	66	-	6.5	0	463	9.2	328	-	11.3	-	1,825	12.8
**Education level**																		
No schooling	112	-	5.9	-	194	2.1	18	-	1.8	-	25	0.5	130	-	4.5	-	219	1.5
Elementary school	943	-	49.8	-	3,304	35.9	299	-	29.3	-	1,069	21.3	1,242	-	42.6	-	4,373	30.8
High school	279	-	14.7	-	1,803	19.6	163	-	16.0	-	1,178	23.5	442	-	15.2	-	2,981	21.0
University education	130	-	6.9	-	1,120	12.2	108	-	10.6	-	957	19.1	238	-	8.2	-	2,077	14.6
Not applicable	138	-	7.3	-	276	3.0	39	-	3.8	-	97	1.9	177	-	6.1	-	373	2.6
Missing data	292	-	15.4	-	2,511	27.3	394	-	38.6	-	1,685	33.6	686	-	23.5	-	4,196	29.5
**Result of the parasitological examination**																		
*P. vivax*	1,585	-	83.7	-	6,299	68.4	740	-	72.5	-	3,204	63.9	2,325	-	79.8	-	9,503	66.8
*P. falciparum*	263	-	13.9	-	2,363	25.7	249	-	24.4	-	1,516	30.3	512	-	17.6	-	3,879	27.3
*P. falciparum* + *P. vivax*	34	-	1.8	-	487	5.3	18	-	1.8	-	219	4.4	52	-	1.8	-	706	5.0
*P. malariae*	9	-	0.5	-	29	0.3	11	-	1.1	-	25	0.5	20	-	0.7	-	54	0.4
*P. ovale*	3	-	0.2	-	23	0.2	2	-	0.2	-	38	0.8	5	-	0.2	-	61	0.4
*P. falciparum* + *P. malariae*	0	-	0.0	-	7	0.1	1	-	0.1	-	9	0.2	1	-	0.0	-	16	0.1

**Legend: N:** Number of cases autochthonous. *Incidence (per 100,000 inhabitants) calculated with the average of the population of each group. -: No data to calculate incidence.

**FIGURE 1:**
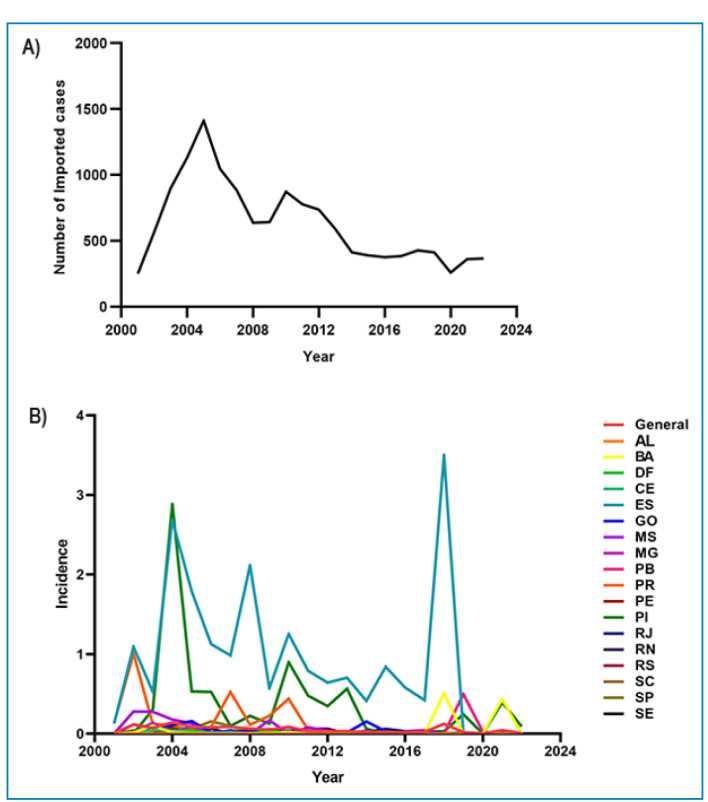
Time series of imported cases and the incidence rate of autochthonous malaria cases in the extra-Amazon region of Brazil.

**Legend: (A)** Number of imported cases per year; **(B)** Incidence of indigenous cases by state and year.

Table 2 indicates that the incidence of autochthonous cases (per 100,000 inhabitants) in the extra-Amazon region ranged from 0.0 to 0.1, with an overall decreasing trend from 2001 to 2022 (APC: -4.6; 95% CI: -8.8 to -0.2). However, the state of PB showed an increasing trend (AAPC: 39.1; 95% CI: 10.3 to 75.3) throughout the study period. Additionally, BA from 2005 to 2022 (APC: 25.1; 95% CI: 8.9 to 43.7), PE from 2005 to 2008 (APC: 109.7; 95% CI: 58.7 to 177.1), and RS from 2001 to 2018 (APC: 6.4; 95% CI: 3.5 to 9.3) exhibited increasing trends in at least one-time interval. The year 2004 recorded the highest number of imported cases, which coincided with the second-highest peak in the incidence of autochthonous cases (Figure 1A). ES maintained the highest rates over the years, yet interestingly, no cases have been recorded there since 2019 (Figure 1B).

**TABLE 2:** Time trends in incidence rates of autochthonous malaria cases by state in the extra-Amazon region of Brazil.

Variables	Period	Incidence rate**	Segmented period APC (95% CI)	Trend	Entire period AAPC (95% CI)	Trend
REA	2001 - 2022	0.0 - 0.1	-4.6* (-8.8 to -0.2)	Decreasing		
AL	-	-	-	-	-	-
BA	2001 - 2005	0.0 - 0.1	-47.7 (-93.1 to 297.5)	Stable	6.0 (-27.1 to 54.0)	Stable
	2005 - 2022	0.5 - 0.0	25.1* (8.9 to 43.7)	Increasing		
CE	2001 - 2022	0.0 - 0.1	-8.4* (-11.1 to -5.6)	Decreasing		
DF	2001 - 2022	0.0 - 0.04	-3.8 (-10.0 to 2.8)	Stable		
ES	2001 - 2004	0.1 - 2.6	72.8 (-43.4 to 427.6)	Stable	62.8 (-)	-
	2004 - 2017	2.6 - 0.4	-11.2* (-17.0 to -5.0)	Decreasing		
	2017 - 2022	3.5 - 0.0	659.1 (-)	-		
GO	2001 - 2022	0.0 - 0.2	-5.6 (-11.4 to 0.5)	Stable		
MS	2001 - 2009	0.0 - 0.3	-10.3* (-16.6 to -3.4)	Decreasing	-18.1* (-26.3 to -9.1)	Decreasing
	2009 - 2022	0.2 - 0.0	-22.6* (-35.1 to -7.7)	Decreasing		
MG	2001 - 2003	0.0 - 0.1	441.5 (-46.4 to 5370.8)	Stable	8.8 (-13.4 to 31.7)	Stable
	2003 - 2022	0.1 - 0.0	-10.0* (-14.7 to -5.1)	Decreasing		
PB	2001 - 2010	0.0 - 0.02	323.8* (212.3 to 475.0)	Increasing	39.1* (10.3 to 75.3)	Increasing
	2010 - 2019	0.4 - 0.0	38.6 (-3.8 to 99.6)	Stable		
	2019 - 2022	0.0 - 0.4	-95* (-98.3 to -85.7)	Decresasing		
PR	2001 - 2022	0.0 - 1.0	-10.5 (-20.6 to 1.0)	Stable		
PE	2001 - 2005	0.0 - 0.1	-94.7 (-100.0 to 930.1)	Stable	-50.0 (-80.2 to 25.9)	Stable
	2005 - 2008	0.04 - 0.0	109.7* (58.7 to 177.1)	Increasing		
	2008 - 2022	0.0 - 0.04	-30.2* (-42.2 to -15.5)	Decreasing		
PI	2001 - 2007	0.0 - 2.9	-35.2 (-66.1 to 23.9)	Stable	-14.3 (-29.3 to 3.8)	Stable
	2007 - 2022	0.9 - 0.0	-4.2 (-15.7 to 9.0)	Stable		
RJ	2001 - 2022	0.0 - 0.1	-2.6 (-7.8 to 2.8)	Stable		
RN	2001 - 2022	0.0 - 0.1	2.6 (-2.7 to 8.3)	Stable		
RS	2001 - 2018	0.0 - 0.03	6.4* (3.5 to 9.3)	Increasing	-18.3 (-68.0 to 108.5)	Stable
	2018 - 2022	0.01 - 0.0	-73.3 (-99.9 to 5203.0)	Stable		
SC	2001 - 2022	0.0 - 0.02	-4.3* (-5.6 to -3.0)	Decreasing		
SP	2001 - 2006	0.0 - 0.2	40.9 (-2.4 to 103.6)	Stable	-6.3 (-14.8 to 3.0)	Stable
	2006 - 2022	0.2 - 0.0	-17.6* (-23.0 to -11.7)	Decreasing		
SE	2001 - 2020	0.0 - 0.1	-51.6* (-63.0 to -36.9)	Decreasing		

**Legend:** *p-value; **Per 100,000 inhabitants; **APC:** Annual Percentage Changes; **AAPC:** Average Annual Percentage Changes; **REA:** Extra-Amazon region; **AL:** Alagoas; **BA:** Bahia; **CE:** Ceará; **DF:** Federal District; **ES:** Espírito Santo; **GO:** Goiás; **MS:** Mato Grosso do Sul; **MG:** Minas Gerais; **PB:** Paraíba; **PR:** Paraná; **PE:** Pernambuco; **PI:** Piauí; **RJ:** Rio de Janeiro; **RN:** Rio Grande do Norte; **RS:** Rio Grande do Sul; **SC:** Santa Catarina; **SP:** São Paulo; **SE:** Sergipe.

**FIGURE 2:**
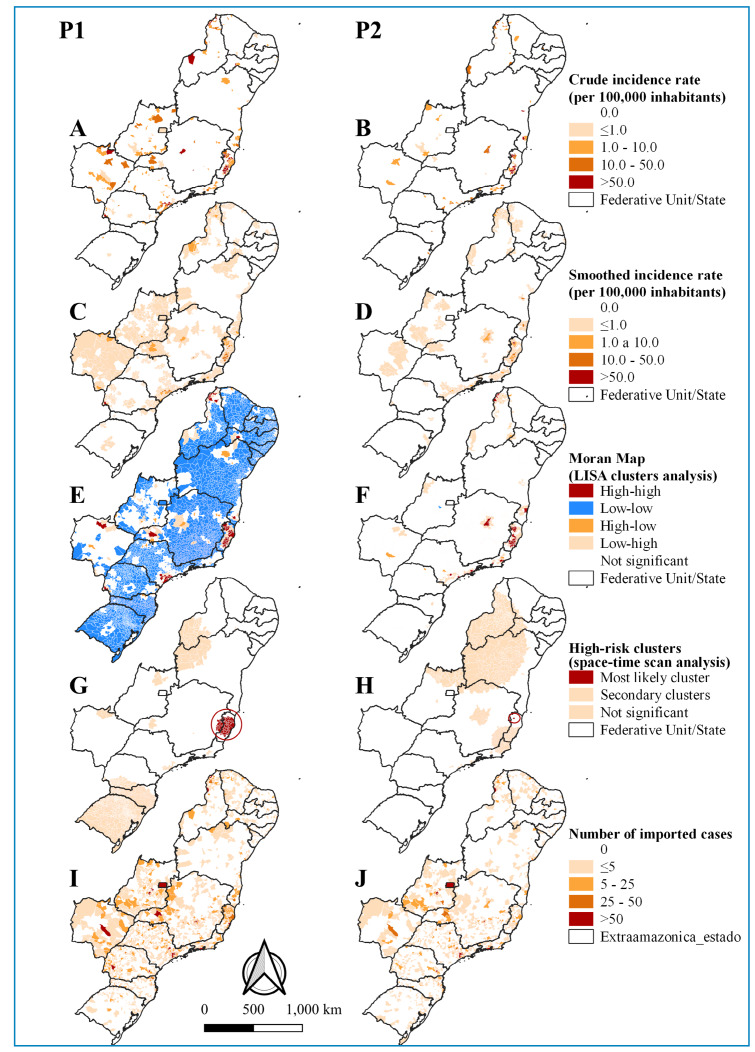
Spatial distribution and spatiotemporal analysis of autochthonous malaria incidence rates, alongside the spatial distribution of imported malaria cases in the extra-Amazon region.

**Legend:** Incidence rate per 100,000 inhabitants; **(P1)** 2001 to 2011; **(P2)** 2012 to 2022; **(A-B)** Crude malaria incidence rate; **(C-D)** Smoothed malaria incidence rate; **(E-F)** Univariate LISA analysis; **(LISA)**. **(G-H)** Spatiotemporal scan analysis. (I-J) Number of imported cases.

The distribution of both crude and smoothed malaria incidence rates was widespread across the majority of federation units in the extra-Amazon region. In P1, high incidence rates were found in the federation units of ES, MG, MS, PE, PI, PR, and SP (25 municipalities), and in P2, in the federation units of BA, ES, MG, PB, and PI (17 municipalities) (Figures 2A-B). Notably, the federation units most affected by malaria in both periods were ES, MG, and PI. Using the Bayesian method, we observed a reduction in high rates and a dispersion of very low rates (Figures 2C-D). 

In the univariate GMI spatial autocorrelation analysis, we identified spatial dependence of malaria cases in municipalities with similar patterns during both periods studied (P1, GMI = 0.106, *p*-value = 0.001; P2, GMI = 0.038, *p*-value = 0.001). Figures 2E-F illustrate that among the federation units with high-risk clusters in P1 (BA, ES, MG, MS, PI, PE, and SC; 56 municipalities), only PE, MS, and SC experienced a decrease in high-risk clusters in P2, with a total of 36 municipalities retaining a high-risk cluster. Conversely, we observed a decrease in low-risk clusters from 3,800 municipalities in P1 to just 2 in P2. Furthermore, there was a shift in the distribution of cases within this region, where low-risk clusters that persisted from P1 transformed into transition zones, and high-risk clusters appeared in other areas of the federation units that were already identified as high risk in P2. 

Using space-time analysis, we identified 17 clusters in P1 (1 to 14: *p*-value = <0.001) and 14 clusters in P2 (1 to 10: *p*-value = <0.001). The primary cluster in P1 included the highest number of cases from 2004 to 2008 (n = 295) located in the states of ES and MG. The annual incidence rate was 2.1 per 100,000 inhabitants, with a RR of 35.60 and a LLR of 731.456158. In P2, the primary cluster comprised 112 cases in a municipality in ES, with an annual incidence rate of 1,216.7 per 100,000 inhabitants, an RR of 45,820.97, and an LLR of 1,078.764358 (Figure 2G-H; Table 3).

**TABLE 3:** Spatiotemporal clusters of the annual malaria incidence rate per 100,000 inhabitants in the extra-Amazon region, Brazil.

P	Clusters	Period	Number of municipalities	States	Number of cases	Expected number of new cases	Annual incidence rate*	RR**	LLR**
P1 (2001 to 2010)	17	2004 - 2008	63	ES, MG	295	10.53	2.1	35.60	731.456158
		2001 - 2002	103	PR	103	0.038	205.3	2,908.70	714.363159
		2004 - 2004	2	PI	79	0.013	455.3	6,329.29	610.08621
		2005 - 2009	8	SP	69	0.97	5.4	74.61	227.696975
		2003 - 2003	1	MG	24	0.0036	510.4	6,800.46	187.581252
		2005 - 2005	3	MG	14	0.033	32.0	423.59	70.642769
		2004 - 2004	1	PE	6	0.0083	55.1	723.98	33.503530
		2006 - 2007	1	SP	59	16.73	0.3	3.64	32.777399
		2006 - 2007	180	RS, SC, PR	44	11.79	0.3	3.83	26.138341
		...	...	...	...	...	...	...	...
P2 (2012 to 2022)	14	2018 - 2018	1	ES	112	0.0030	1,216.7	45,820.97	1078.764358
		2018 - 2018	1	BA	75	0.0069	351.5	12,357.58	626.821877
		2013 - 2017	193	ES, MG, RJ	134	10.35	0.4	16.16	232.679569
		2019 - 2019	1	PB	20	0.0080	81.1	2,595.73	136.917159
		2012 - 2013	3	PI	18	0.019	30.2	963.64	105.430905
		2012 - 2012	1	SP	11	0.016	21.6	682.32	60.699991
		2016 - 2019	5	SP	19	0.43	1.4	45.33	53.606256
		2016 - 2017	27	MG	13	0.25	1.7	53.58	38.866561
		2017 - 2017	1	RJ	6	0.096	2.0	62.77	18.904705
		...	...	...	...	...	...	...	...

**Legend: P:** segmented period; **RR:** relative risk for the cluster compared to the rest of the region; **LLR:** likelihood ratio; *Malaria incidence rate per 100,000 inhabitants during the cluster period. **Relative risk and likelihood ratio with significant p-value.

The distribution of the absolute number of imported cases was noted in all federation units of the extra-Amazon region. The highest number of cases was observed in 9 municipalities in CE, DF, GO, MG, PI, and PR during P1. Conversely, in P2, the highest number of cases was recorded in 19 municipalities across the federation units of CE, DF, GO, MG, PI, PR, RJ, SC, and SP (Figure 2I-J).

## DISCUSSION

In supporting Brazil's goal to eliminate malaria by 2035, our analysis across two study periods (2001 to 2011 and 2012 to 2022) revealed that males, aged 20-30 and 40-59 years, and those of indigenous descent consistently showed the highest incidence rates. Individuals with primary education and diagnosed with*P. vivax*constituted the majority of cases. Notably, there was an increase in the percentage of*P. falciparum*diagnoses from P1 to P2 (13.0% to 24.4%). This rise may be linked to cases entering from French Guiana, primarily due to the migration of individuals involved in illegal mining activities in that region^20^. Additionally, non-endemic regions such as the United States, United Kingdom, and Italy also reported significant increases in*P. falciparum*malaria cases among travelers, with rates of 60.0%, 0.73%, and 4.5% respectively^21^.

The temporal trend was stable for most states, yet there was an observable decline in the overall incidence within the extra-Amazon region in recent years. Similarly, our spatial analysis indicated a reduction in the number of municipalities classified as high risk. 

Our findings are in agreement with similar reports developed in the extra-Amazon region from 2007 to 2014^7^ and 2011 to 2020^22^, which also identified males and the economically active age groups of 20 to 59 as having the highest incidence rates. However, regarding race/color, our results mirrored those from the Amazon region, emphasizing the challenges that indigenous populations face in accessing health services, including preventive measures and appropriate treatment^23^. The education factor appears to be linked to the migration of these individuals to endemic areas for employment, increasing their exposure to vectors during field activities ^24^.

In other American countries, as well as in the rest of Brazil,*P. vivax*is the species responsible for the majority of malaria cases^25^. However, in non-endemic areas such as the extra-Amazon region of Brazil, febrile symptoms are often mistakenly attributed to dengue due to the lack of qualified professionals capable of accurately diagnosing the species, consequently affecting proper treatment. For instance, in RJ, three patients were incorrectly diagnosed with dengue; tragically, one of them died ^26^. Therefore, the actual number of*P. vivax*cases and related deaths in this region may be underreported^27^.

The occurrence of malaria in the extra-Amazon region, particularly in urban centers, raises significant concerns due to the conducive conditions for the spread of the parasite and vector in densely populated areas^6^ ^,^ ^8^. Although most cases in this region are imported, primarily linked to travel and tourism from states like Amazonas and Rondônia, as well as from Africa^5^ ^,^ ^28^, the occurrence of autochthonous cases serves as a critical alert for the surveillance system. These cases heighten the risk of community transmission and the potential establishment of malaria^29^, especially in areas with diverse vector populations such as Brazil^5^ ^,^ ^6^ ^,^ ^30^.

Furthermore, a study by Wetzler and colleagues (2022) revealed that imported cases have surged since 2018 in the state of Roraima, predominantly among workers arriving from Venezuela and Guyana^31^. These authors also noted that most autochthonous cases are linked to mining activities in the state's endemic areas. However, the likelihood of imported cases among miners is higher, indicating that unregulated illegal mining in indigenous territories is a primary contributor to these cases. This data strongly suggests that the source of infection for both imported and autochthonous cases is the same, exacerbated by the constant movement of individuals between endemic and non-endemic regions^31^.

 It is crucial to note the occurrence of simian malaria in Brazil. Simian malaria, which affects non-human primates, was widely reported across various regions of Brazil in 1992, parasitizing wild primates and suggesting potential transmission to humans^32^. Similarly, in Malaysia, many patients were erroneously diagnosed with*P. malariae*when in fact the infections were caused by other*Plasmodium*species, a mistake that could also occur in Brazil due to the similar morphological characteristics among species^33^. 

Historically, several factors have contributed to malaria outbreaks in the extra-Amazon region, including tourism and travel-related cases, natural disasters^34^, and ecological changes due to human activities, primarily in civil construction^35^. Additionally, geographic and environmental factors, particularly in states within the Atlantic Forest region, affect the distribution of autochthonous malaria cases by providing favorable conditions for vector spread^6^. This may explain the persistence of high-risk clusters in ES, MG, PI, SC, SP, and PR. Another contributing factor could be the influx of cases from northern Brazil into GO^36^, and similarly, the border location of MS with GO and SP facilitates the movement of infected individuals, increasing the risk of disease transmission in these non-endemic areas^37^.

Our findings indicate a reduction in the number of malaria cases in the extra-Amazonian region during the two periods analyzed. Adding to this, Ferreira and Castro have documented a decline in malaria prevalence over the years, with the disease being nearly eliminated from the Northeast, Southeast, and South regions of Brazil, and transmission mostly contained in the Central-West, excluding the Amazon basin^38^. 

The Central-Western region of Brazil experiences high incidence rates of malaria, potentially due to its proximity to the Legal Amazon, which facilitates the dispersion of disease vectors. Notably, the state of Mato Grosso, located in this region, encompasses the central-southern portion of the Brazilian Amazon, an area historically known for high malaria incidence^39^. Additionally, during the 1990s, the states of Goiás (in the Extra-Amazon region) and Mato Grosso (in the Legal Amazon region) reported the highest rates of disability-adjusted life years (DALYs). The continued presence of malaria in this region suggests favorable conditions for parasite transmission^36^.

It is significant that the number of municipalities with very high malaria incidence rates for malaria decreased in P2 compared to P1. However, there was an expansion in municipalities with low incidence rates, especially after the smoothing of rates, indicating factors that support the maintenance of the*Plasmodium spp.*cycle at low levels, particularly in states and municipalities bordering the Amazon basin. Moreover, autochthonous cases of malaria occur, albeit in small proportions, in states within the Atlantic Forest biome: ES, MG, SP, RJ, SC, and PR. The occurrence of malaria in these Atlantic Forest states underscores the possibility of focal transmission, potentially involving non-human primates in the transmission cycle^38^. This could explain the persistence of infection rates in regions traditionally considered non-endemic for malaria.

Despite the presence of some high-risk clusters, our study indicates a decrease in such clusters between the two periods examined, with low-risk clusters playing a crucial role in malaria transmission. We demonstrated that when low-risk clusters are not eliminated, they promote the development of transition zones and may even lead to the emergence of high-risk clusters, underscoring the need for control and surveillance not only in areas traditionally perceived as high-risk. Therefore, for Brazil to meet the objectives of the National Malaria Elimination Plan by 2035, adherence to recommendations for low-risk areas must be stringent. This approach should include mandatory and immediate case notification, individual monitoring, supervised treatment, and location-specific actions aimed at preventing an increase in cases and further reducing incidence rates. Additionally, effective control of surveillance and health measures targeting the mosquito vector is essential^40^.

The limitations of this study relate to the use of secondary data, which may lead to underreporting or overreporting of malaria cases in certain areas, or errors in diagnosis that could impact the accurate characterization of the sociodemographic aspects of the population. Additionally, reliance on secondary data assumes the accuracy and completeness of official records, potentially introducing biases that are challenging to control. Given that this is an ecological study, the group-level data presented may not accurately reflect individual occurrences. Nevertheless, our findings elucidate the temporal, spatial, and spatiotemporal dynamics of malaria over the past 22 years, providing critical insights for decision-making in combating malaria in Brazil and identifying key demographic groups and regions to target in order to achieve the goals of the National Malaria Elimination Plan. 

## CONCLUSION

Our findings reveal a decrease in malaria cases across two study periods, a declining trend, and a reduction in high-risk clusters within the extra-Amazon region of Brazil. Conversely, most federation units exhibited a stable trend, and some low-risk clusters evolved into transitional or high-risk areas. This epidemiological situation underscores malaria as a significant public health issue in Brazil, emphasizing the challenges health services face in meeting the objectives of the National Malaria Elimination Plan. The authors also caution against the risk of malaria transmission in states traditionally considered non-endemic and illustrate that even though the federation units are outside the Amazon region, autochthonous cases heighten the risk of community transmission and the persistence of malaria. Therefore, to effectively manage malaria in the extra-Amazon region, enhanced diagnostic and treatment strategies are essential to mitigate the risk of disease outbreaks. Additionally, the implementation of the plan’s strategies must be stringent and tailored to the specific needs of each population.
