# Supplementary material for: Sociodemographic aspects, time series and high-risk clusters of malaria in the extra-Amazon region of Brazil: a 22-year study
Source: Rev Soc Bras Med Trop. 2024 Nov 8;57:e00421-2024. doi: 10.1590/0037-8682-0564-2023 (PMC11656533; doi:10.1590/0037-8682-0564-2023)
Supplement: Supplementary file 1 [file 1678-9849-rsbmt-57-e00421-2024-supp1.pdf]

## Supplementary Material 1.

| | NUMBER OF AUTOCHTONOUS CASES PER FEDERATION UNIT AND PER YEAR | | | | | | | | | | | | | | | | | | | | | |
|---|---|---|---|---|---|---|---|---|---|---|---|---|---|---|---|---|---|---|---|---|---|---|
| FU | 2001 | 2002 | 2003 | 2004 | 2005 | 2006 | 2007 | 2008 | 2009 | 2010 | 2011 | 2012 | 2013 | 2014 | 2015 | 2016 | 2017 | 2018 | 2019 | 2020 | 2021 | 2022 |
| PI | 0 | 0 | 9 | 86 | 16 | 16 | 3 | 7 | 4 | 28 | 15 | 11 | 18 | 2 | 0 | 0 | 1 | 1 | 8 | 0 | 13 | 3 |
| CE | 0 | 0 | 4 | 3 | 3 | 1 | 2 | 0 | 0 | 0 | 0 | 0 | 0 | 1 | 0 | 0 | 0 | 0 | 0 | 0 | 0 | 1 |
| RN | 0 | 0 | 0 | 0 | 1 | 2 | 0 | 0 | 0 | 1 | 2 | 2 | 0 | 0 | 0 | 0 | 0 | 0 | 0 | 0 | 0 | 0 |
| PB | 0 | 0 | 0 | 0 | 0 | 0 | 0 | 0 | 0 | 1 | 0 | 0 | 0 | 0 | 0 | 0 | 0 | 0 | 20 | 1 | 0 | 0 |
| PE | 0 | 0 | 0 | 7 | 0 | 1 | 1 | 4 | 2 | 2 | 0 | 0 | 0 | 0 | 0 | 0 | 0 | 0 | 0 | 0 | 0 | 0 |
| AL | 0 | 0 | 0 | 0 | 0 | 0 | 0 | 0 | 0 | 0 | 0 | 0 | 0 | 0 | 0 | 0 | 0 | 0 | 0 | 0 | 0 | 0 |
| SE | 0 | 0 | 0 | 1 | 0 | 0 | 0 | 0 | 0 | 0 | 0 | 0 | 0 | 0 | 0 | 0 | 0 | 0 | 0 | 0 | 0 | 0 |
| BA | 0 | 2 | 14 | 2 | 1 | 0 | 0 | 1 | 1 | 3 | 0 | 1 | 0 | 0 | 0 | 1 | 0 | 75 | 0 | 0 | 65 | 0 |
| MG | 1 | 3 | 25 | 2 | 18 | 0 | 0 | 0 | 0 | 0 | 0 | 0 | 0 | 0 | 0 | 5 | 9 | 0 | 0 | 0 | 1 | 1 |
| ES | 4 | 35 | 17 | 90 | 61 | 39 | 33 | 73 | 20 | 44 | 28 | 23 | 27 | 16 | 33 | 23 | 17 | 139 | 0 | 0 | 0 | 0 |
| RJ | 0 | 3 | 11 | 3 | 4 | 1 | 2 | 4 | 3 | 6 | 0 | 2 | 4 | 4 | 10 | 5 | 7 | 1 | 1 | 1 | 0 | 1 |
| SP | 1 | 17 | 14 | 8 | 25 | 64 | 39 | 7 | 26 | 8 | 2 | 11 | 10 | 1 | 0 | 4 | 8 | 4 | 7 | 2 | 5 | 4 |
| PR | 13 | 99 | 12 | 11 | 4 | 9 | 54 | 12 | 24 | 46 | 5 | 1 | 0 | 0 | 0 | 0 | 0 | 1 | 0 | 0 | 0 | 0 |
| RS | 1 | 0 | 0 | 0 | 0 | 0 | 0 | 0 | 0 | 2 | 0 | 0 | 0 | 0 | 0 | 0 | 0 | 0 | 1 | 0 | 0 | 0 |
| SC | 0 | 2 | 0 | 0 | 0 | 0 | 0 | 0 | 0 | 0 | 0 | 0 | 0 | 0 | 0 | 0 | 0 | 0 | 0 | 1 | 0 | 0 |
| MS | 0 | 6 | 6 | 4 | 3 | 0 | 1 | 0 | 4 | 0 | 2 | 1 | 0 | 1 | 0 | 0 | 0 | 0 | 0 | 0 | 0 | 0 |
| GO | 0 | 6 | 4 | 6 | 9 | 1 | 2 | 1 | 3 | 5 | 2 | 0 | 1 | 10 | 2 | 0 | 1 | 0 | 0 | 0 | 1 | 1 |
| DF | 0 | 0 | 0 | 0 | 1 | 0 | 0 | 0 | 1 | 0 | 0 | 0 | 0 | 0 | 0 | 0 | 0 | 0 | 0 | 0 | 0 | 0 |
