# Supplementary material for: Sociodemographic aspects, time series and high-risk clusters of malaria in the extra-Amazon region of Brazil: a 22-year study
Source: Rev Soc Bras Med Trop. 2024 Nov 8;57:e00421-2024. doi: 10.1590/0037-8682-0564-2023 (PMC11656533; doi:10.1590/0037-8682-0564-2023)
Supplement: Supplementary file 2 [file 1678-9849-rsbmt-57-e00421-2024-supp2.pdf]

## Supplementary Material 2.

| | NUMBER OF IMPORTED CASES PER FEDERATION UNIT AND PER YEAR | | | | | | | | | | | | | | | | | | | | | |
|---|---|---|---|---|---|---|---|---|---|---|---|---|---|---|---|---|---|---|---|---|---|---|
| FU | 2001 | 2002 | 2003 | 2004 | 2005 | 2006 | 2007 | 2008 | 2009 | 2010 | 2011 | 2012 | 2013 | 2014 | 2015 | 2016 | 2017 | 2018 | 2019 | 2020 | 2021 | 2022 |
| PI | 0 | 5 | 16 | 53 | 50 | 78 | 30 | 31 | 33 | 57 | 69 | 53 | 48 | 24 | 10 | 17 | 12 | 17 | 8 | 5 | 19 | 19 |
| CE | 0 | 1 | 40 | 55 | 67 | 40 | 49 | 26 | 22 | 47 | 28 | 21 | 19 | 10 | 9 | 16 | 9 | 13 | 16 | 11 | 19 | 10 |
| RN | 11 | 7 | 12 | 8 | 13 | 13 | 5 | 7 | 12 | 13 | 11 | 16 | 5 | 7 | 8 | 2 | 8 | 5 | 8 | 0 | 1 | 3 |
| PB | 1 | 3 | 5 | 2 | 12 | 4 | 1 | 1 | 10 | 16 | 8 | 10 | 4 | 0 | 8 | 3 | 1 | 3 | 4 | 1 | 5 | 1 |
| PE | 3 | 2 | 11 | 17 | 17 | 8 | 26 | 19 | 29 | 15 | 19 | 8 | 20 | 14 | 11 | 6 | 4 | 6 | 4 | 6 | 4 | 6 |
| AL | 0 | 2 | 7 | 9 | 5 | 2 | 5 | 4 | 8 | 4 | 5 | 8 | 7 | 5 | 3 | 2 | 7 | 1 | 1 | 0 | 3 | 0 |
| SE | 0 | 1 | 9 | 2 | 6 | 2 | 2 | 1 | 4 | 7 | 7 | 6 | 2 | 5 | 4 | 3 | 1 | 5 | 1 | 1 | 2 | 2 |
| BA | 3 | 12 | 44 | 27 | 38 | 28 | 29 | 25 | 18 | 22 | 18 | 12 | 14 | 15 | 12 | 16 | 7 | 11 | 9 | 7 | 12 | 21 |
| MG | 52 | 86 | 83 | 149 | 167 | 124 | 111 | 96 | 83 | 116 | 105 | 92 | 83 | 32 | 35 | 52 | 58 | 39 | 46 | 27 | 23 | 22 |
| ES | 13 | 46 | 66 | 77 | 101 | 68 | 54 | 48 | 31 | 32 | 38 | 24 | 23 | 17 | 20 | 23 | 29 | 11 | 22 | 0 | 0 | 1 |
| RJ | 2 | 23 | 52 | 68 | 92 | 87 | 61 | 62 | 60 | 63 | 82 | 118 | 64 | 50 | 55 | 36 | 41 | 48 | 39 | 24 | 27 | 26 |
| SP | 61 | 134 | 170 | 213 | 320 | 224 | 183 | 123 | 133 | 204 | 175 | 154 | 126 | 111 | 97 | 106 | 93 | 111 | 95 | 61 | 76 | 75 |
| PR | 29 | 60 | 119 | 132 | 171 | 116 | 122 | 67 | 79 | 56 | 69 | 50 | 46 | 29 | 22 | 15 | 20 | 43 | 36 | 21 | 32 | 29 |
| RS | 24 | 32 | 45 | 58 | 67 | 49 | 44 | 23 | 20 | 42 | 31 | 40 | 40 | 31 | 18 | 19 | 25 | 29 | 38 | 22 | 29 | 23 |
| SC | 6 | 10 | 15 | 18 | 20 | 16 | 11 | 6 | 5 | 18 | 7 | 10 | 6 | 12 | 6 | 2 | 6 | 11 | 4 | 6 | 7 | 9 |
| MS | 0 | 27 | 45 | 41 | 72 | 51 | 25 | 24 | 28 | 22 | 22 | 22 | 13 | 4 | 3 | 4 | 11 | 5 | 10 | 8 | 14 | 11 |
| GO | 46 | 118 | 155 | 162 | 142 | 94 | 92 | 55 | 43 | 89 | 54 | 72 | 46 | 34 | 46 | 38 | 24 | 52 | 56 | 52 | 75 | 86 |
| DF | 0 | 0 | 3 | 42 | 52 | 43 | 34 | 19 | 25 | 50 | 30 | 21 | 24 | 12 | 22 | 16 | 28 | 18 | 15 | 7 | 12 | 21 |
